# Induction of interferon signaling and allograft inflammatory factor 1 in macrophages in a mouse model of breast cancer metastases

**DOI:** 10.12688/wellcomeopenres.16569.2

**Published:** 2021-06-23

**Authors:** Wei Zheng, Dejian Zhao, Hui Zhang, Prameladevi Chinnasamy, Nicholas Sibinga, Jeffrey W. Pollard

**Affiliations:** 1 Department of Developmental and Molecular Biology, Albert Einstein College of Medicine and Montefiore Medical Center, Bronx, New York, 10461, USA; 2 Division of Hematology and Oncology, Icahn School of Medicine at Mount Sinai, New York, New York, 10029, USA; 3 Yale Center for Genome Analysis, Yale University, New Haven, Connecticut, 06510, USA; 4 MRC Centre for Reproductive Health, Queen’s Medical Research Institute, The University of Edinburgh, Edinburgh, EH16 4TJ, UK

**Keywords:** breast cancer, metastasis, macrophage, interferon, AIF1, IBA1, tumor microenvironment

## Abstract

**Background:** Metastatic breast cancer cells recruit macrophages (metastasis-associated macrophages, or MAMs) to facilitate their seeding, survival and outgrowth. However, a comprehensive understanding of the gene expression program in MAMs and how this program contributes to metastasis remain elusive.

**Methods:** We compared the transcriptomes of MAMs recruited to lung metastases and resident alveolar macrophages (RAMs) and identified a large variety of differentially expressed genes and their associated signaling pathways. Some of the changes were validated using qRT-PCR and immunofluorescence. To probe the functional relevance to metastatic growth, a gene-targeting mouse model of female mice in the C57BL6/J background was used to study allograft inflammatory factor 1 (AIF1, also known as ionized calcium-binding adapter molecule 1 or IBA1).

**Results:** Interferon signaling is one of the most activated pathways in MAMs, with strong upregulation of multiple components of the pathway and a significant enrichment for the gene signatures of interferon-alpha-treated human macrophages. *Aif1*, an interferon-responsive gene that regulates multiple macrophage activities, was robustly induced in MAMs. *Aif1* deficiency in MAMs, however, did not affect development of lung metastases, suggesting that AIF1 indicates MAM activation but is dispensable for regulating metastasis.

**Conclusions:** The drastically different gene expression profile of MAMs as compared to RAMs suggests an important role in promoting metastatic growth. Dissection of the underlying mechanisms and functional validation of potential targets in the profile may provide novel therapeutic strategies for the treatment of metastatic diseases.

## Abbreviations

AIF1: allograft inflammatory factor 1; BM: bone marrow; BMT: bone marrow transplantation; FACS: fluorescence-activated cell sorting; GSEA: gene set enrichment analysis; IFN: interferon; iv: intravenous; IPA: ingenuity pathway analysis; IVIS: *in vivo* imaging system; MAMs: metastasis-associated macrophages; RAMs: resident alveolar macrophages; RNA-seq: RNA-sequencing; WT: wild type.

## Introduction

Tumor-associated macrophages (TAMs) are important in multiple steps of tumorigenesis and progression, including regulation of tumor cell invasion into the stroma, intravasation into blood vessels, seeding at distal organs, angiogenesis, inflammation and immune suppression ( Qian & Pollard, 2010). Compared to the abundant knowledge of tumor-associated macrophages at primary sites, the role of the macrophages at metastatic sites, i.e. metastasis-associated macrophages (MAMs), has only begun to emerge in recent years. In mice, lung metastases resulting from mammary cancer have been shown to recruit MAMs that are CD11B ^+^CD11C ^-^, as compared to CD11B ^-^CD11C ^+^ resident alveolar macrophages (RAMs) ( Qian *et al.*, 2009). MAMs and their monocytic progenitors express CCR2 and respond to the CCL2 signal emitted from metastases ( Lu & Kang, 2009; Qian *et al.*, 2011). Once recruited to the metastatic site, MAMs can secrete vascular endothelial growth factor A to facilitate cancer cell extravasation for seeding of the lung ( Qian *et al.*, 2011). MAMs also secrete CCL3, which acts in an autocrine manner to augment MAM recruitment and enhance their activity to promote metastatic growth ( Kitamura *et al.*, 2015). Despite the significance of these findings, they represent only a patchwork of a global program of MAMs, which remains elusive. To this end, we performed deep RNA sequencing (RNA-seq) in MAMs isolated from micro-dissected lung metastases 11 days after tumor cell inoculation to gain unbiased insight into the global transcriptional program and to learn how MAMs may use this program to regulate metastatic growth in secondary organs.

## Methods

### Ethics statement and animal experiments

All procedures involving mice were conducted in accordance with National Institutes of Health regulations concerning the use and care of experimental animals and were approved by the Albert Einstein College of Medicine Animal Use Committee (animal protocol numbers: 20180202 and 20180205). Mice were housed and maintained in a barrier facility at the Albert Einstein College of Medicine and were kept on 12-hour light/dark cycle and had ad libitum access to chow and water. The number of animals used in each experiment is explained in figure legends. A total of 66 mice were used. No mice were excluded from analyses. All efforts were made to ameliorate harm to animals. Mice carrying lung metastases were terminated by cervical dislocation after anesthesia with isoflurane before showing signs of difficulty breathing. Anesthesia with isoflurane was also performed for intravital imaging as below.

C57BL6/J wild type (WT) mice were purchased from the Jackson Laboratory and were pooled together and were randomly assigned to the non-treated control group or the metastasis group. Lung metastases were induced by intravenous (iv) injection of 1 × 10 ^6^ E0771-LG cells ( Kitamura *et al.*, 2015) to the tail vein of syngenic C57BL6/J female mice (6–8 weeks old) and were analyzed on day 11. *Aif1* -/- mice were obtained by replacing all coding sequences with a modified LacZ gene in the 129SvEv background and were backcrossed into the C57BL6/J background for 14 generations by NS ( Casimiro *et al.*, 2013) and derived from his colony according to genotype.

For bone marrow transplantation (BMT), total bone marrow (BM) cells were extracted from 8–12 weeks old WT or *Aif1* -/- female donor mice from their femurs, tibiae and spine by grinding in a mortar. Red blood cells were removed by incubation with RBC Lysis Buffer (Biolegend 420301) for 5 min at 4°C prior to injections. Female WT recipient mice of 4–6 weeks old were irradiated with 10 Gy gamma rays split into two doses with a four-hour interval (Mark I-68A 137Cs irradiator from JL Shepherd and Associates, San Fernando, CA). The random allocation of mice to experimental group (WT vs *Aif1*-/-) was driven by Mendelian Inheritance. The mice were then iv injected with 1 × 10 ^7^ BM cells from one of the donors the next day. Five weeks after BMT, lung metastases were induced by iv injection of 1 × 10 ^6^ E0771-LG-luciferase-ZsGreen cells ( Zheng *et al.*, 2019), and the mice were analyzed on day 10 by *in vivo* imaging system (IVIS) and histology.

For IVIS imaging, mice were anesthetized with isoflurane (2% v/v with oxygen as the carrier gas) in an inhalation chamber (VetEquipt 911103) before retro-orbital injection with d-luciferin in PBS (GoldBio, LUCK-1G, 1.5 mg/100 µL/20 g mouse). Afterwards, the mice were imaged using IVIS Spectrum In Vivo Imaging System (Perkin Elmer) while the anesthesia was maintained in the imaging chamber. Photon flux (photon/second/cm ^2^/steradian) in the lung area was analyzed using Living Image Software (Perkin Elmer, v4.3.1; a possible free alternative is Aura Imaging Software from Spectral Instruments Imaging) and was expressed as relative values with respect to the lowest value in the WT group.

### Flow cytometry and cell sorting

Lungs were perfused with PBS through the right ventricle, and metastases (<1 mm in diameter) were dissected under a dissection microscope and pooled from 1–5 mice. The tissues were subsequently digested using an enzyme mix of Liberase DL (Sigma-Aldrich 5466202001, 0.52 U/mL), TL (Sigma-Aldrich 5401020001, 0.26 U/mL) and DNase I (Sigma-Aldrich DN25, 150 µg/mL) for 30 min at 37°C. For sorting for RNA-seq or qRT-PCR, transcription inhibitors alpha-amanitin (Sigma-Aldrich A2263, 5 µg/mL) and actinomycin D (Sigma-Aldrich A1410, 1 µg/mL) were also added to the digestion buffer. Flow cytometry was analyzed using a LSRII cytometer (BD Biosciences), and the data were analyzed using Flowjo software (TreeStar, v10; a possible free alternative is Flowing Software 2.5.1 from University of Turku). FACSAria II (BD Biosciences) and Moflo Astrios (Beckman Coulter) were used for sorting. Antibody information is found in Table 1. 

**Table 1.** Antibodies used for flow cytometry, immunofluorescence and immunohistochemistry.

Antibodies	Source	Identifier	Dilutions (µL/100 µL)
AIF1	Abcam	Cat# ab178847	0.3
Anti-rabbit-ImmPRESS-HRP	Vector Laboratories	Cat#MP-6401-15	Ready-to-use
Anti-rat-ImmPRESS-HRP	Vector Laboratories	Cat#MP-7444, RRID:AB_2336530	Ready-to-use
CD11B, FITC, clone M1/70	BD Biosciences	Cat# 561688, RRID:AB_10898180	0.5
CD11B, PE-Cy7, clone M1/70	BioLegend	Cat# 101215, RRID:AB_312798	0.5
CD11B, PE, clone M1/70	BD Biosciences	Cat# 561689, RRID:AB_10893803	0.5
CD11C, BUV395, clone HL3	BD Biosciences	Cat# 564080, RRID:AB_2738580	1
CD11C, FITC, clone HL3	BD Biosciences	Cat# 561045, RRID:AB_10562385	1
CD45, APC-Fire750, clone 30-F11	BioLegend	Cat# 103153, RRID:AB_2572115	0.8
CD45, APC, clone 30-F11	BD Biosciences	Cat# 559864, RRID:AB_398672	0.8
CD45, PerCP-Cy5.5, clone 30-F11	BD Biosciences	Cat# 550994, RRID:AB_394003	0.8
Donkey anti-rabbit, AF594	ThermoFisher	Cat# A-21207, RRID:AB_141637	0.3
Donkey anti-rat-biotin	Jackson ImmunoResearch	Cat# 712-065-153	0.2
Donkey anti-rat, AF488	ThermoFisher	Cat# A-21208, RRID:AB_2535794	0.3
F4/80, AF647, clone Cl:A3-1	Bio-Rad	Cat# MCA497A647, RRID:AB_323931	6
LY6C, BV421, clone AL-21	BD Biosciences	Cat# 562727, RRID:AB_2737748	0.5
MRC1, PE-Cy7, clone C068C2	BioLegend	Cat# 141719, RRID:AB_2562247	1
MRC1, PE, clone C068C2	Biolegend	Cat# 141705, RRID:AB_10896421	1
Streptavidin, AF488	Jackson ImmunoResearch	Cat# 016-540-084	0.2

### RNA isolation, qRT-PCR and RNA sequencing

Total RNAs were extracted using Picopure RNA Isolation kit (Arcturus KIT0202). RNA sequencing was performed at Beijing Genomic Institute, using the Ovation® RNA-Seq System V2 kit for library construction, pair-end 100 bp and Hiseq 4000, which generated 60–80M reads per sample. The reads were aligned to the mouse reference genome (GRCm38/mm10) using Tophat (v2.0.13) ( Kim *et al.*, 2013). Uniquely mapped reads were counted for each gene using htseq-count in the HTSeq package (v0.6.1) with gene models from UCSC RefGene ( Anders *et al.*, 2015). FPKM values were generated using Cufflinks (v2.2.1) ( Trapnell *et al.*, 2013). Differentially expressed genes were identified using DESeq2 ( Love *et al.*, 2014). For qRT-PCR, RNAs were reverse transcribed and amplified using QuantiTect® Whole Transcriptome (Qiagen 207043) before qPCR. Gene expression was normalized to *beta-actin*. Relative expression is calculated using the formula –ddCt, where Ct stands for threshold cycles. See Table 2 for Taqman gene expression assays.

**Table 2.** Information for the Taqman probes.

Gene	Taqman assay ID
Actb	Mm02619580_g1
Aif1	Mm01132451_g1
Ifi35	Mm01260550_g1
Ifit1	Mm00515153_m1
Ifitm2	Mm00850080_g1
Ifitm3	Mm00847057_s1
Ifnar2	Mm00494916_m1
Irf1	Mm01288580_m1
Irf9	Mm00492679_m1
Isg15	Mm01705338_s1
Stat1	Mm01257286_m1
Stat2	Mm00490880_m1
Tap1	Mm00443188_m1

### Unsupervised hierarchical clustering, Gene Ontology, canonical pathway enrichment and gene set enrichment analysis

**Figure 1.**
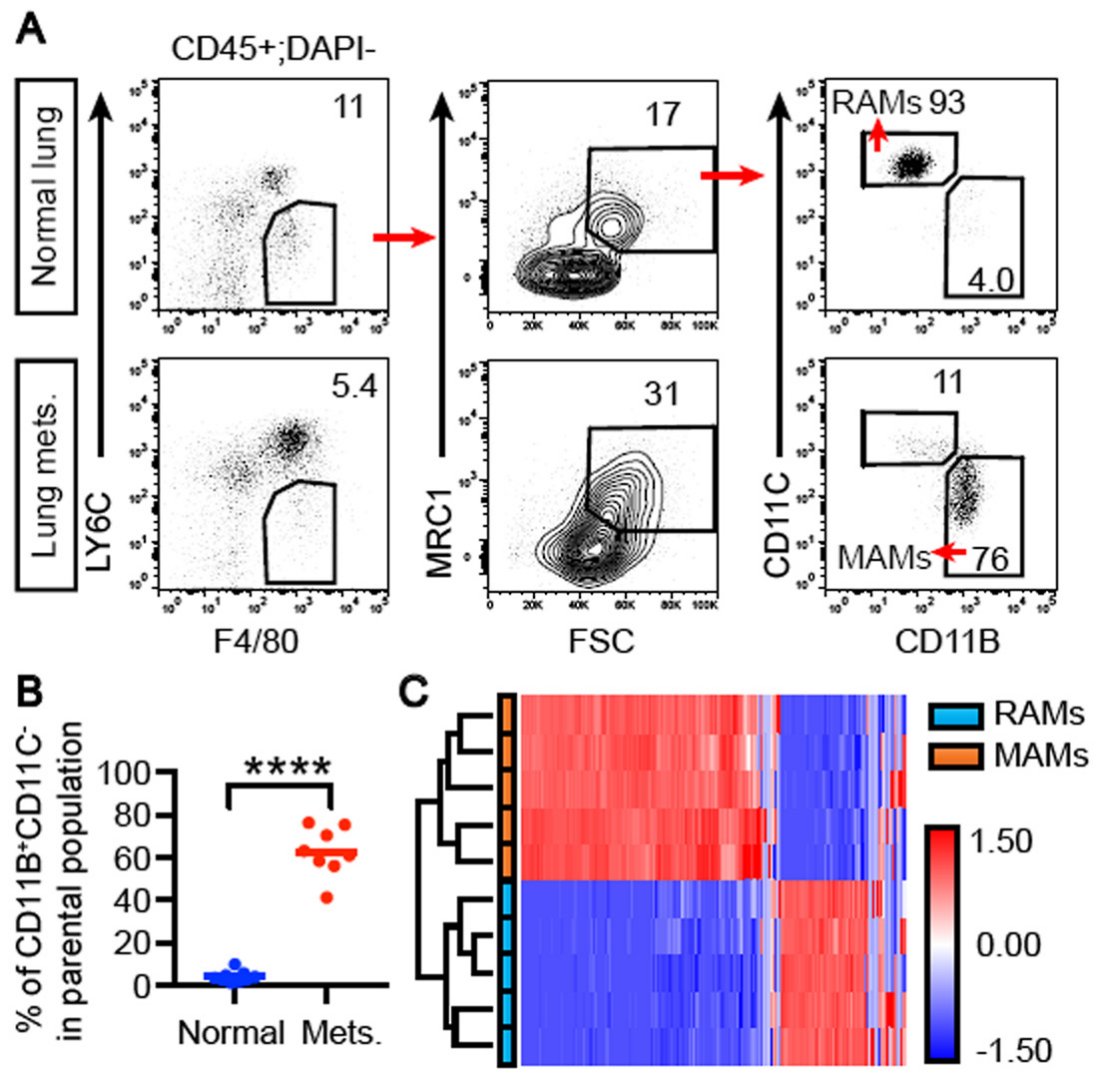
Metastasis-associated macrophages (MAMs) recruited to lung metastases express a distinct gene expression program. ( **A**) Gating strategy for isolating MAMs and resident alveolar macrophages (RAMs). Lung metastases (mets.) were induced by intravenous injection of E0771-LG cells, dissected and prepared for fluorescence-activated cell sorting (FACS) sorting for MAMs. RAMs from normal lungs were sorted similarly. ( **B**) Quantification of the CD11B ^+^CD11C ^-^ population in panel A. Pool of three independent experiments including one for the qRT-PCR analysis in Figure 2B. N = 9 control mice and eight metastases samples pooled from 18 mice in total. ****, p <0.0001. ( **C**) Sorted MAMs and RAMs were analyzed with RNA-sequencing, and the gene expression dataset was analyzed using unsupervised hierarchical clustering.

For unsupervised hierarchical clustering, FPKM values were transformed by log2. Without prior grouping, genes of log2(mean of FPKMs) > 5 and standard deviation > 1.5 were used for clustering. The differentially expressed genes (Extended Data: Table 3 ( Zheng *et al.*, 2021)) were analyzed for gene ontology annotation using the DAVID software (v6.8) ( Huang *et al.*, 2009) and the canonical pathway analysis using QIAGEN Ingenuity Pathway Analysis (QIAGEN IPA, March 2017 release; a possible free alternative is DAVID v6.8) ( Krämer *et al.*, 2014). For gene set enrichment analysis (GSEA), the MAM gene expression data were pre-ranked by the false-discovery rate (FDR) values: for upregulated genes, -log2 (FDR + A); for downregulated genes, log2 (FDR + A), and A = minimal FDR value that is not 0. This pre-ranked gene list was then analyzed in GSEA to interrogate if the three gene sets associated with IFN-alpha treatment of human macrophages ( Greenwell-Wild *et al.*, 2009; Liu *et al.*, 2012; Tassiulas *et al.*, 2004) as indicated in Figure 1 showed significant enrichment. GSEA (v3.0) was used for the analysis ( Subramanian *et al.*, 2005).

### Immunofluorescence and immunohistochemistry

Briefly, lungs were perfused with 1% w/v PFA through the right ventricle, inflated with 2% w/v agarose in PBS via the trachea (for frozen sections) or 1% w/v PFA (for paraffin sections), followed by immersion fixation in 4% w/v PFA for 1 hour at +4°C. For frozen embedding, the tissues were incubated in 25% w/v sucrose in PBS for 6 h or overnight before embedding in OCT Compound (Fisher HealthCare, 4585). Staining was performed in a similar manner to that previously published ( Zheng *et al.*, 2019). Briefly, 20-µm frozen sections or 5-µm paraffin sections of the lung were permeabilized with PBS containing 0.3% v/v Triton-X, blocked with PBS containing 0.3% v/v Triton-X, 5% v/v donkey serum, 0.05% w/v sodium azide and 0.2% w/v bovine serum albumin, and incubated with primary antibodies overnight. The sections were subsequently washed and incubated with secondary antibodies conjugated with fluorochromes or horseradish peroxidase (HRP). For HRP-conjugated secondary antibodies, the signal was visualized using ImmPACT® NovaRED® Substrate (Vector Laboratories, SK-4805). For F4/80 immunofluorescence, an additional amplification step using anti-rat-biotin and streptavidin-AF488 was performed. For antigen retrieval in immunohistochemistry of paraffin sections, Tris-EDTA (pH 9.0) (VWR, #K043) for AIF1 was used. See Table 1 for the antibody details.

Quantification of lung metastases was performed similarly as previously described ( Nielsen *et al.*, 2001). Briefly, paraffin sections were stained with hematoxylin and scanned with the High Capacity Slide Scanner PANNORAMIC 250 Flash III (3D Histech). Images were analyzed with Fiji (NIH, v2.0.0-rc-64/1.51s), using the Colour Deconvolution and Analyze Particles functions, to quantify the total area of lung metastases and the total lung area. Eighteen mice (nine in each group) were analyzed for histology of lung metastases.

### Statistical analysis

Each sample represents an individual mouse or pooled lung metastases obtained from 1–5 mice. Student’s t test or two-way ANOVA (for pooling of multiple experiments) were used. Statistical analyses were carried out with Graphpad Prism (version 7). For RNA-seq analysis, multiple hypothesis testing was adjusted using the Benjamini and Hochberg FDR method. Sample sizes were determined empirically.

## Results

### Gene expression profiling identifies strong activation of interferon signaling in MAMs

To analyze the gene expression profiles of MAMs and RAMs, we dissected metastases from affected lungs 11 days after seeding and normal lungs before cell dissociation for fluorescence-activated cell sorting (FACS) sorting of MAMs and RAMs. Consistent with previous findings, ( Kitamura *et al.*, 2015; Qian *et al.*, 2009; Qian *et al.*, 2011), lung metastases were significantly enriched with the CD11B ^+^CD11C ^-^ subset of CD45 ^+^F4/80 ^+^LY6C ^-^MRC1 ^+^FSC ^high^ macrophages, as compared to the predominant CD11B ^-^CD11C ^+^ RAMs in normal lungs ( Figure 1A and 1B). RNA-seq of these two populations revealed distinct gene expression programs between the two, as unsupervised hierarchical clustering completely segregated the two populations ( Figure 1C).

**Figure 2.**
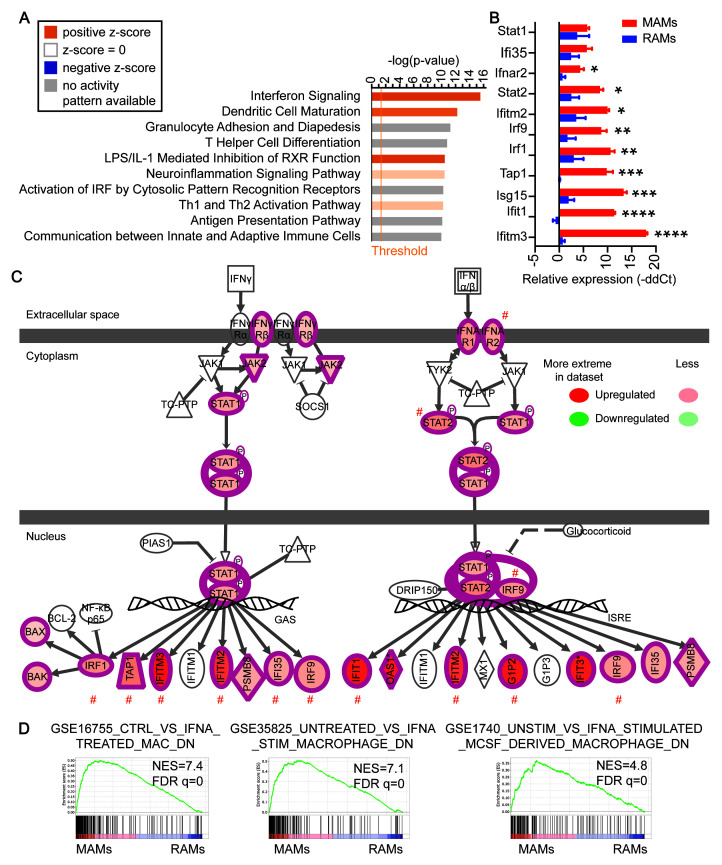
Activation of the interferon pathway in metastasis-associated macrophages (MAMs). ( **A**) Top 10 canonical pathways enriched by the differentially expressed genes in MAMs as analyzed using Ingenuity Pathway Analysis (IPA). ( **B**) qRT-PCR analysis of resident alveolar macrophages (RAMs) and MAMs for genes in the Interferon Signaling pathway. Mean +/- standard error of the mean N = 3 control mice and three lung metastasis samples pooled from eight mice. ****, p < 0.0001; ***, p < 0.001; **, p < 0.01; *, p <0.05. ( **C**) Schematic representation of the activation of the Interferon Signaling pathway. Shades of redness indicate the extent of upregulation as detected by the RNA-sequencing. #, significantly upregulated genes as validated in panel B are marked here. ( **D**) Gene set enrichment analysis showed enrichments of three gene sets from IFN-alpha-treated human macrophages in MAMs, indicating a strong interferon signature in MAMs compared to RAMs.

Gene Ontology annotation of the genes differentially expressed by MAMs showed enrichment for cellular responses to cytokine stimulus and reduction in lipid metabolism (Extended Data: Tables 1–3 ( Zheng *et al.*, 2021)). To study the biological pathways enriched by the differentially expressed genes in more detail, we performed canonical pathway enrichment analysis using QIAGEN IPA (Extended Data: Table 4 ( Zheng *et al.*, 2021)). “Interferon (IFN) Signaling pathway” was the most enriched pathway and was predicted to be significantly activated ( Figure 2A). An independent qRT-PCR analysis of the genes in this pathway confirmed the upregulation of most of the genes ( Figure 2B and 2C). In addition, GSEA revealed that MAMs were strongly enriched for three independent gene sets obtained from IFN-alpha-treated human macrophages ( Figure 2D). These data suggest that increased expression of the pathway components indeed translates into the signaling output of the pathway. Together with the additional significantly altered pathways such as the NF-kappa B pathway and various interleukin pathways (Extended Data: Table 5 ( Zheng *et al.*, 2021)), these findings suggest that MAMs recruited to lung metastases have a profoundly different gene expression program from RAMs indictive of unique functional roles.

### AIF1 expression indicates MAM activation but is dispensable for regulation of lung metastases

**Figure 3.**
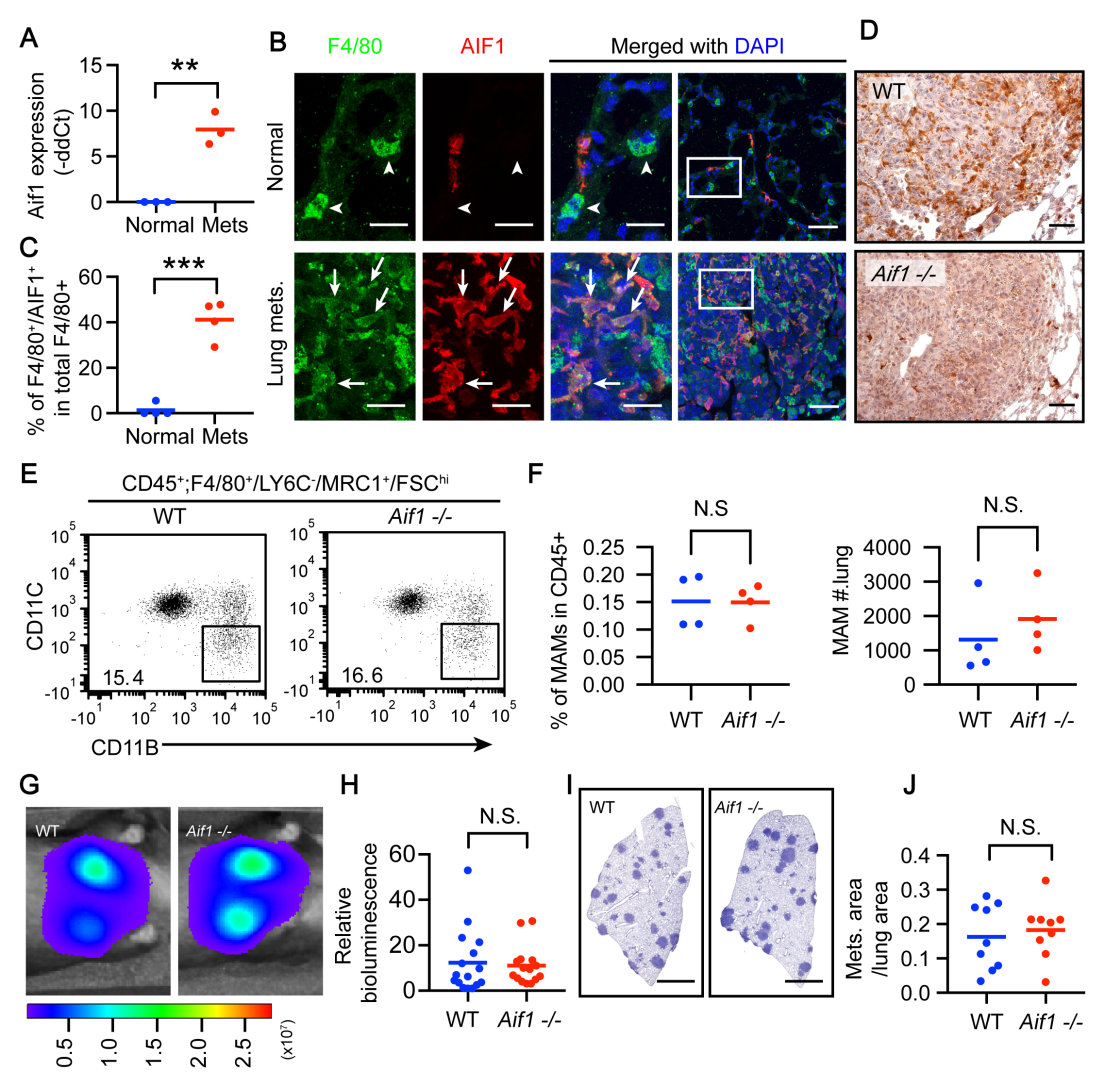
AIF expression is induced in metastasis-associated macrophages (MAMs) but is dispensable for regulation of metastatic growth in the lung. ( **A**) qRT-PCR analysis of *Aif1* in resident alveolar macrophages (RAMs) and MAMs. The same mice and samples were used as in Figure 2B. **, p <0.01. ( **B**) Immunofluorescent staining of lung sections from normal mice and mice bearing lung metastases induced by E0771-LG intravenous (iv) injection. Arrowheads, F4/80 ^+^ macrophages negative for AIF1. Arrows, F4/80 ^+^ macrophages positive for AIF1. White boxes in column 4 are magnified in columns 1–3. Scale bars = 20 µm in columns 1-3 and 50 µm in column 4. ( **C**) Quantification of F4/80 ^+^AIF1 ^+^ cells in total F4/80 ^+^ cells in panel B. N = 4 control mice and 4 metastasis-bearing mice. ***, p < 0.001. ( **D**) Immunohistochemistry for AIF1 of the lung sections from panel F. Dark brown indicates the positive signal. Scale bars = 50 µm. ( **E**) Lethally irradiated wild-type (WT) recipient mice that received bone marrow transplantation from WT or *Aif1* -/- mice were injected iv with E0771-LG. Total lung cells were analyzed for MAM recruitment to lung metastases 10 days after the injection. ( **F**) Quantification of panel E. N = 4 mice in each group. ( **G**) The mice in panel E and additional mice with the same treatments were also analyzed for metastatic growth using *in vivo* imaging system imaging. ( **H**) Quantification of the bioluminescent signal from panel G. Pool of two experiments. Total N = 16 WT mice and 15 *Aif1* -/- mice pooled from two independent experiments. N.S., not significant. ( **I**) Hematoxylin staining of lung sections from mice in panel G. Scale bars = 500 µm. ( **J**) Quantification of the total area of metastatic nodules per mouse normalized to total lung area in panel F. N = 9 mice in each group pooled from two independent experiments. N.S., not significant.

To study if and how genes differentially expressed by MAMs may regulate lung metastasis, we focused on *Aif1*. It is one of the most upregulated genes (Log _2_(fold change)>6, Extended Data: Table 3 ( Zheng *et al.*, 2021)) and is in two significantly enriched gene sets that are relevant to the current study – “response to interferon-gamma” and “leukocyte chemotaxis” (Extended Data: Table 4 ( Zheng *et al.*, 2021), GO:0034341 and GO:0030595). AIF1 is an intracellular calcium-binding and IFN-gamma-inducible protein mostly expressed in the monocyte/macrophage lineage ( Zhao *et al.*, 2013). It integrates various inflammatory stimuli, supports production of particular cytokines, and is critical for survival and pro-inflammatory activity of macrophages ( Chinnasamy *et al.*, 2015; Zhao *et al.*, 2013). We verified with an independent qRT-PCR assay the dramatic upregulation of the *Aif1* transcript in MAMs ( Figure 3A). This finding was also corroborated by immunofluorescent staining for the AIF1 protein, which showed that approximately 40% of macrophages within metastatic nodules robustly expressed AIF1, whereas little was detected in RAMs in the normal lung ( Figure 3B and 3C).

Next, we tested if induction of AIF1 in MAMs is required for promoting metastatic growth in the lung. To restrict *Aif1* gene ablation to the hematopoietic lineage, we performed a bone marrow transplantation assay in which the bone marrow of *Aif1* -/- donor mice ( Casimiro *et al.*, 2013) was transplanted into WT recipient mice. Lung metastases were induced in these mice for comparison with WT-to-WT recipient mice. The efficiency of BM engraftment of *Aif1* ^-/-^ cells is confirmed by the marked decrease of AIF1+ cells in the lung metastases ( Figure 3D). However, *Aif1* deficiency did not impair MAM recruitment to lung metastases ( Figure 3E and F). Nor did it affect the MAM function in regulation of metastatic growth, as evaluated by both bioluminescence and histology ( Figure 3G–J). Together, these data suggest that AIF1 indicates MAM activation, but its expression by MAMs is dispensable for regulation of metastasis.

## Discussion

In this study, we profiled the transcriptome of MAMs by deep RNA-seq and identified a distinct expression program that are indicative of important metastasis-regulatory functions.

IFN signaling has been shown to have anti-metastatic effects via inhibition of epithelial-to-mesenchymal transition of tumor cells, angiogenesis, intravasation, survival in the circulation, homing to target tissues, and extravasation ( Ortiz & Fuchs, 2017). The effects of IFN treatment on MAMs with respect to regulation of metastasis are, however, unknown. Given that ablation of MAMs or inhibition of MAM recruitment suppressed metastatic growth in multiple models ( Lu & Kang, 2009; Nielsen *et al.*, 2016; Qian *et al.*, 2011; Qian *et al.*, 2009), it is plausible that activation of IFN signaling promotes the pro-metastatic activity of MAMs, which may therefore limit the favorable effects of IFN-alpha treatment for patients of metastatic diseases ( Eggermont *et al.*, 2014). In support of this hypothesis, STAT1, an essential signaling molecule of the IFN pathway, is found to be induced in TAMs and mediate suppression of T cell response ( Kusmartsev & Gabrilovich, 2005). It has been suggested that the difficulty to target the right IFNs at the right dose in the right type of cells might underlie the basis for the limited therapeutic effects of IFNs ( Dunn *et al.*, 2006). It is thus interesting to determine in future work if macrophage-specific inactivation of IFN signaling inhibits development of metastasis, and more importantly if MAM-targeting will improve IFN therapy and immunotherapy.

Despite robust induction of the interferon-inducible gene *Aif1* in MAMs, we did not see an effect of AIF1 deficiency on development of lung metastases. Although we cannot rule out the possibility that the residual host-derived *Aif1* WT BM cells may be sufficient to rescue the impaired function of *Aif1*-/- cells (if any), it is a less likely event given the magnitude of reduction of AIF1-expressing cells. Another possible reason is that AIF1 may play opposite roles in different cell types, and thus the constitutive gene deletion may result in a counterbalance. Since bone marrow transplantation has restricted the effects to the hematopoietic lineage, and *Aif1* is highly expressed by monocytes/macrophages ( Zhao *et al.*, 2013), the possibility of counterbalancing is minimal. Nevertheless, a macrophage-specific deletion of *Aif1* in future studies will clarify this issue. A similar strong correlation of AIF1-expressing macrophages/microglial cells with malignancy has been previously observed for human gliomas ( Deininger *et al.*, 2000). Another study shows that AIF1 is expressed by brain-infiltrating myeloid cells that enhance metastatic growth by promoting proliferation and reducing apoptosis of tumor cells ( Zhang *et al.*, 2015). Functional testing in neither study has been attempted. It is thus interesting to determine whether AIF1 expression in macrophages or microglia plays a functional role in these settings or serves as a marker that indicates cell reprogramming in the tumor microenvironment, as identified in our study.

It is interesting to see if the mouse data can extend to the human setting. However, samples of patient lung metastases are not readily accessible. Comparison with human TAMs at the primary site ( Cassetta *et al.*, 2019) reveals that some general pathways are shared between human TAMs and mouse MAMs, such as cytokine production and leukocyte activation. However, there are also specific effects. For example, the IFN pathway activation only exists in mouse MAMs. Organ-specific effects are not surprising, as tumor cells are under different selective pressures, and the contextual cues in the microenvironment that affect tumor-regulating stromal cells are also distinct ( Joyce & Pollard, 2009; Nguyen *et al.*, 2009). The latter notion is exemplified by two recent findings. First, while DKK1 expressed by cancer cells inhibits lung metastasis, it promotes bone metastasis due to the respective non-canonical and canonical WNT signaling in these two organs ( Zhuang *et al.*, 2017). Second, an atypical type of large platelets accumulate only in lung metastases but not in mammary tumors. Only those accumulated in lung metastases but not circulating ones express high levels of cytokines, which regulate a variety of myeloid cell functions ( Zheng *et al.*, 2019).

In conclusion, we have identified a distinct gene expression program in MAMs that include strong activation of the IFN pathway and AIF1. Since cancer cells at metastatic sites benefit from these recruited MAMs, the gene expression profile revealed in this study can provide an important platform to uncover novel mechanisms and potential therapeutic targets that are essential for MAMs to promote metastatic growth. 

## Data availability

### Underlying data

Gene Expression Omnibus: RNA-seq data on NCBI Gene Expression Omnibus. Accession number GSE124933; https://identifiers.org/geo:GSE124933.

Open Science Framework: Raw data for "Induction of interferon signaling and allograft inflammatory factor 1 in macrophages in a mouse model of breast cancer metastases". 10.17605/OSF.IO/42T98 ( Zheng, 2021).

This project contains the following underlying data:

- Fig. 1A and B: FCS files for the FACS data and the values for the graph in panel B in XLSX format.
- Fig. 2B: ddCt values for the qPCR analysis of the IFN pathway.
- Fig. 3A: Ct values for the *Aif1* qPCR analysis.
- Fig. 3B and C: Raw images of the fluorescence and values for the graph.
- Fig. 3D: Raw images for AIF1 immunohistochemistry.
- Fig. 3E and F: Raw FCS files for the flow cytometric analysis and values for the graph.
- Fig. 3G and H: Raw IVIS images and values for the graph.
- Fig. 3I and J: Raw images for histological evaluation of lung metastases and values for the graph.

Data are available under the terms of the Creative Commons Zero "No rights reserved" data waiver (CC0 1.0 Public domain dedication).

### Extended data

Figshare: Extended Data Tables for "Induction of interferon signaling and allograft inflammatory factor 1 in macrophages in a mouse model of breast cancer metastases". https://doi.org/10.6084/m9.figshare.13567940.v1 ( Zheng *et al.*, 2021).

This project contains the following extended data:

1. **Extended Data Table 1:** **Fragments per kilobase of transcript per million mapped reads (FPKM) values of MAMs and RAMs.** Uniquely mapped reads were counted for each gene using htseq-count in the HTSeq package (v0.6.1) with gene models from UCSC RefGene.
2. **Extended Data Table 2: Differentially expressed genes as identified using DEseq2.** Mean FPKM values < 1 in both groups under comparison were excluded from differential expression analysis. Fold change >2, FDR < 0.05.
3. **Extended Data Table 3: Highly differentially expressed genes for IPA analysis.** This gene set is obtained with the intersection of Extended Data Table 1 and 2, which is subsequently applied with the following more stringent criteria: log2 (fold change) > 1.5, FDR < 0.03; for upregulated genes, mean FPKMs of MAMs > 20; for downregulated genes, mean FPKMs of RAMs > 40.
4. **Extended Data Table 4: Gene Ontology annotation of the highly differentially expressed genes.** Upregulated and downregulated genes from Extended Data Table 3 were analyzed by the GOBP5 analysis of DAVID Bioinformatics Resources.
5. **Extended Data Table 5: Canonical Pathways in IPA enriched by the differentially expressed genes in MAMs.** Output for the Canonical Pathways in IPA using the highly differentially expressed genes from Extended Data Table 3.

Data are available under the terms of the Creative Commons Attribution 4.0 International license (CC-BY 4.0).
