# Supplementary material for: Induction of interferon signaling and allograft inflammatory factor 1 in macrophages in a mouse model of breast cancer metastases
Source: Wellcome Open Res. 2021 Jun 23;6:52. Originally published 2021 Mar 8. [Version 2] doi: 10.12688/wellcomeopenres.16569.2 (PMC8008350; doi:10.12688/wellcomeopenres.16569.2)
Supplement: Peer review history

# Peer review: Reviewer response for version 2

**DOI:** 10.21956/wellcomeopenres.18757.r44618

**Published:** 2021-07-05

**Authors:** Keehoon Jung

**Affiliations:** 1 Department of Biomedical Sciences, Department of Anatomy and Cell Biology, Seoul National University College of Medicine, Seoul, South Korea

The authors have adequately addressed my previous questions in the revision. I have no further queries, and I think the revised manuscript is acceptable for publication.

Is the work clearly and accurately presented and does it cite the current literature?

Yes

If applicable, is the statistical analysis and its interpretation appropriate?

Yes

Are all the source data underlying the results available to ensure full reproducibility?

Yes

Is the study design appropriate and is the work technically sound?

Yes

Are the conclusions drawn adequately supported by the results?

Yes

Are sufficient details of methods and analysis provided to allow replication by others?

Yes

Reviewer Expertise:

cancer immunology, myeloid cells, intravital microscopy, single-cell genomics

I confirm that I have read this submission and believe that I have an appropriate level of expertise to confirm that it is of an acceptable scientific standard.

# Peer review: Reviewer response for version 1

**DOI:** 10.21956/wellcomeopenres.18259.r42975

**Published:** 2021-03-25

**Authors:** Keehoon Jung

**Affiliations:** 1 Department of Biomedical Sciences, Department of Anatomy and Cell Biology, Seoul National University College of Medicine, Seoul, South Korea

This manuscript titled “Induction of interferon signaling and allograft inflammatory factor 1 in macrophages in a mouse model of breast cancer metastases” by Zheng et al. compares upregulated signaling pathways in MAMs (metastasis-associated macrophages) to those in RAMs (resident alveolar macrophages). The authors performed transcriptomic analyses that highlight the difference of macrophages in lung metastasis from those in normal lung, which led to evaluate the effect of targeting MAM-Aif1 in lung metastases. Although Aif1 deficiency in MAMs does not seem to be really important in lung metastasis, unfortunately, I believe this set of results needs to be shared by the research community to improve the understanding in the field. I only have some minor comments as summarized below:

1. To study the effect of Aif1 deficiency in MAMs, the authors performed a series of BMT of WT and Aif1-/- mice to WT recipient mice, respectively. If conditional Aif1 knockout mouse model was available, it would have been clearer to more specifically target Aif1 only in macrophages. Arent there any Aif1 cKO mice available, and if not/so the authors could discuss a bit in the discussion section?
2. In Figure 3, the authors represent histological evaluation of the lung metastases of WT-to-WT mice and Aif1-/--to-WT mice which shows the lack of Aif1 expression in metastatic nodules of Aif1-/--to-WT group. In addition to this, have the authors analyzed (or counted) macrophage infiltration per se to lung metastases between these two groups by any chance?
3. In Results, the authors may want to provide relevant reference(s) to support the following statement: “Consistent with previous findings, lung metastases were significantly enriched with the CD11B ^+^CD11C ^- ^subset of CD45 ^+^F4/80 ^+^LY6C ^-^MRC1 ^+^FSC ^high^ macrophages, as compared to the predominant CD11B ^-^CD11C ^+^RAMs in normal lungs (Figure 1A and 1B).

Is the work clearly and accurately presented and does it cite the current literature?

Yes

If applicable, is the statistical analysis and its interpretation appropriate?

Yes

Are all the source data underlying the results available to ensure full reproducibility?

Yes

Is the study design appropriate and is the work technically sound?

Yes

Are the conclusions drawn adequately supported by the results?

Yes

Are sufficient details of methods and analysis provided to allow replication by others?

Yes

Reviewer Expertise:

cancer immunology, intravital microscopy, single-cell genomics

I confirm that I have read this submission and believe that I have an appropriate level of expertise to confirm that it is of an acceptable scientific standard.

# Response

**Published:** 2021-06-17

**Authors:** Jeffrey Pollard

**Affiliations:** College of Medicine and Veterinary Medicine, University of Edinburgh, UK

*1. To study the effect of Aif1 deficiency in MAMs, the authors performed a series of BMT of WT and Aif1-/- mice to WT recipient mice, respectively. If conditional Aif1 knockout mouse model was available, it would have been clearer to more specifically target Aif1 only in macrophages. Aren’t there any Aif1 cKO mice available, and if not/so the authors could discuss a bit in the discussion section?*

**Response**: This is a great point. We have generated *Aif1* f/f mice and were indeed planning to cross with Cd169-Cre mice to restrict to macrophages, as indicated by the reviewer. However, since we did not observe a phenotype of metastases even with constitutive gene deletion, we did not move ahead with the conditional knockout. We have modified the Discussion as follows:

 "Another possible reason is that Aif1 may play opposite roles in different cell types, and thus the constitutive gene deletion may result in a counterbalance. Since bone marrow transplantation has restricted the effects to the hematopoietic lineage, and Aif1 is highly expressed by monocytes/macrophages (Zhao et al., 2013), the possibility of counterbalancing is minimal. Nevertheless, a macrophage-specific deletion of Aif1 in future studies will clarify this issue."

*2. In Figure 3, the authors represent histological evaluation of the lung metastases of WT-to-WT mice and Aif1-/--to-WT mice which shows the lack of Aif1 expression in metastatic nodules of Aif1-/--to-WT group. In addition to this, have the authors analyzed (or counted) macrophage infiltration per se to lung metastases between these two groups by any chance?*

***Response**: Again, an excellent point. Unfortunately, as the F4/80 staining in paraffin sections has not been consistent (note that the F4/80 staining in original Fig. 3B was in frozen sections), we were not able to draw any conclusions. However, we have added new data in the revised version, which showed that Aif1 gene ablation did not affect MAM recruitment to lung metastases by flow cytometry (new Fig. 3E and F).*

*3. In the Results, the authors may want to provide relevant reference(s) to support the following statement: “Consistent with previous findings, lung metastases were significantly enriched with the CD11B ^+^CD11C ^- ^subset of CD45 ^+^F4/80 ^+^LY6C ^-^MRC1 ^+^FSC ^high^ macrophages, as compared to the predominant CD11B ^-^CD11C ^+^RAMs in normal lungs (Figure 1A and 1B).*

**Response**: We apologize for the missing references, which are (Kitamura et al., 2015; Qian et al., 2011; 2009). We have updated them in the new version.

# Peer review: Reviewer response for version 1

**DOI:** 10.21956/wellcomeopenres.18259.r42977

**Published:** 2021-03-24

**Authors:** Frances Balkwill

**Affiliations:** 1 Barts Cancer Institute, Queen Mary University of London, London, UK

This short paper presents RNAseq data from lung metastases-associated macrophages – MAMs and resident alveolar macrophages – RAMs – in a mouse cancer model. Having found significant differences in the gene expression patterns between the two populations, and confirmed some of the key results with qRT-PCR and immunofluorescence, the authors focused on the relevance of IFN-signaling pathways and in particular the IFN-induced protein AIF-1. There were very good and validated reasons why this molecule was selected for further investigation but *Aif1 *deficiency in MAMs did not affect the development of lung metastases.

 The most useful aspect of this paper is the RNAseq data from the MAMs and RAMs which will be of interest and use to other researchers. The data are well described and the paper is easy to understand although the results are preliminary.

 Although there are some useful points in the discussion, it would be interesting to include some comment on similarities between the transcriptional profile of the murine MAMs and that of the human tumour-associated macrophages from breast and endometrial cancer profiled in the group’s 2019 paper in Cancer Cell.

 In summary, the data provided can be considered preliminary but are worthy of further investigation.

Is the work clearly and accurately presented and does it cite the current literature?

Partly

If applicable, is the statistical analysis and its interpretation appropriate?

Yes

Are all the source data underlying the results available to ensure full reproducibility?

Yes

Is the study design appropriate and is the work technically sound?

Yes

Are the conclusions drawn adequately supported by the results?

Yes

Are sufficient details of methods and analysis provided to allow replication by others?

Yes

Reviewer Expertise:

NA

I confirm that I have read this submission and believe that I have an appropriate level of expertise to confirm that it is of an acceptable scientific standard, however I have significant reservations, as outlined above.

# Response

**Published:** 2021-06-17

**Authors:** Jeffrey Pollard

**Affiliations:** College of Medicine and Veterinary Medicine, University of Edinburgh, UK

We thank the reviewer for the overall positive comments. It is an excellent point to put the mouse data in the context of human disease. However, the human data we published in Cancer Cell in 2019 were from tumor-associated macrophages (TAMs) in primary tumors. Given the organ-specific effects, interpretations of such a comparison are not always straightforward. Nevertheless, we agree that we should still discuss the similarities and differences and thus have modified the Discussion as follows:

*It is interesting to see if the mouse data can extend to the human setting. However, samples of patient lung metastases are not readily accessible. Comparison with human TAMs at the primary site (Cassetta et al., 2019) reveals that some general pathways are shared between human TAMs and mouse MAMs, such as cytokine production and leukocyte activation. However, there are also specific effects. For example, *the *IFN pathway activation only exists in mouse MAMs. Organ-specific effects are not surprising, as tumor cells are under different selective pressures, and the contextual cues in the microenvironment that affect tumor-regulating stromal cells are also distinct* *(Joyce and Pollard, 2009; Nguyen et al., 2009). This notion is exemplified by two recent findings. First, while DKK1 expressed by cancer cells inhibits lung metastasis, it promotes bone metastasis due to the respective non-canonical and canonical WNT signaling in these two organs (Zhuang et al., 2017). Second, an atypical type of large platelets accumulate only in lung metastases but not in mammary tumors. Only those accumulated in lung metastases but not circulating ones express high levels of cytokines, which regulate a variety of myeloid cell functions (Zheng et al., 2019).*

# Peer review: Reviewer response for version 1

**DOI:** 10.21956/wellcomeopenres.18259.r42976

**Published:** 2021-03-23

**Authors:** Vincenzo Bronte

**Affiliations:** 1 Section of Immunology, Department of Medicine, University of Verona, Verona, Italy

**Brief summary of the main findings**

 The manuscript from Wei Zhang *et al.* describes a gene signature in metastasis associated macrophages (MAMs) isolated from lung nodules of transplantable mouse tumors and compared to resident alveolar macrophages. The interferon (IFN) signaling pathway was highly enriched in MAMs and overlapped with the signature of human macrophages exposed to type I IFN, i.e. IFN-alpha. Among the top regulated genes, allograft inflammatory factor 1 (AIF1, also known as ionized calcium-binding adapter molecule 1 or IBA1) was further investigated. Chimeric mice transplanted with *Aif1*-deficient bone marrow cells did not show differences in the metastatic burden, indicating that AIF1 is not involved in regulating the pro-metastasis activity of MAMs.

**Overall evaluation**

 Even though this manuscript contains a limited number of data, the intriguing observation that type I IFN might have a detrimental effect on tumor immunity is worthy of consideration, especially in view of the limited efficacy of IFN treatment for melanoma patients. These data suggest that the variability in clinical findings might be related to the confounding effects of type I IFN on the metastatic niche.

**Limits of the work and suggestions**

 The current work should be expanded to define whether the IFN signature is also shared by human MAMs, an approach that could also offer the perspective of prioritizing other genes of the IFN-dependent signature and establish the next candidate(s) for in vivo validation studies. However, before fully discarding AIF1, the Authors should confirm molecularly the absence of the gene in MAMs from chimeric mice since the protein detection by immunohistochemistry might not detect a low but functionally relevant amount of AIF1. Finally, experiments in immunodeficient mice will be necessary to sort out the contribution of the IFN-regulated pathway in MAMs independently of their potential effect on adaptive immunity.

Is the work clearly and accurately presented and does it cite the current literature?

Yes

If applicable, is the statistical analysis and its interpretation appropriate?

Yes

Are all the source data underlying the results available to ensure full reproducibility?

Yes

Is the study design appropriate and is the work technically sound?

Yes

Are the conclusions drawn adequately supported by the results?

Yes

Are sufficient details of methods and analysis provided to allow replication by others?

Yes

Reviewer Expertise:

Tumor immunology, innate immunity, tumor escape, immunotherapy, Covid-19 immunopathology

I confirm that I have read this submission and believe that I have an appropriate level of expertise to confirm that it is of an acceptable scientific standard.

# Response

**Published:** 2021-06-17

**Authors:** Jeffrey Pollard

**Affiliations:** College of Medicine and Veterinary Medicine, University of Edinburgh, UK

- We thank the reviewer for the overall positive comments and have addressed the suggestions as below.
- On AIF1. For clarification, the experiment setup was a bone marrow transplantation from constitutive *Aif1*-/- mice to wild-type recipient mice. Thus, the successfully engrafted donor-derived BM cells would have had no expression of AIF1 at all, regardless of the detection method. However, there is a concern that the engraftment might not have been 100% and that some residual host-derived *Aif1* WT cells remained. Indeed, there are a few remaining AIF1+ cells in the lung metastases, but they are much less than in the WT-to-WT control (Original Fig. 3H). We have included the following in Discussion (3 ^rd^ paragraph) to take into account this possibility: “Although we cannot rule out the possibility that the residual host-derived *Aif1* WT BM cells may be sufficient to rescue the impaired function of *Aif1*-/- cells (if any), it is a less likely event given the magnitude of reduction of AIF1-expressing cells. 
- On the contribution of the IFN pathway from different cell types. We agree with the reviewer that IFN signaling acts on a broad range of cell types. To this end, we restricted IFN signaling inactivation to macrophages by crossing *Ifnar1* ^f/f^ mice with *Cd169*-Cre mice. Unfortunately, as the lab was closed, we did not obtain the desired genotype to test this hypothesis before the close-down.
